# Supplementary material for: Alpha2 Adrenergic Modulation of Spike-Wave Epilepsy: Experimental Study of Pro-Epileptic and Sedative Effects of Dexmedetomidine
Source: Int J Mol Sci. 2023 May 29;24(11):9445. doi: 10.3390/ijms24119445 (PMC10254047; doi:10.3390/ijms24119445)
Supplement: Supplementary file 1 [file ijms-24-09445-s001.zip › Supplementary File S1 - SWD detection.pdf]

Supplementary S1
Detection of epileptic spike-wave discharges (SWDs) in the full length epidural EEG

Typical SWDs in rats with genetic predisposition to absence epilepsy appear spontaneously as a sequence of high voltage spikes and waves with a frequency of 8–10 Hz, Figure S1.1 [1,2]. In frequency domain, SWDs have two powerful components: around 8–10 Hz and 16–10 Hz (1st harmonic). These two frequency bands were used for the automatic detection of SWDs (Figure S1.2).

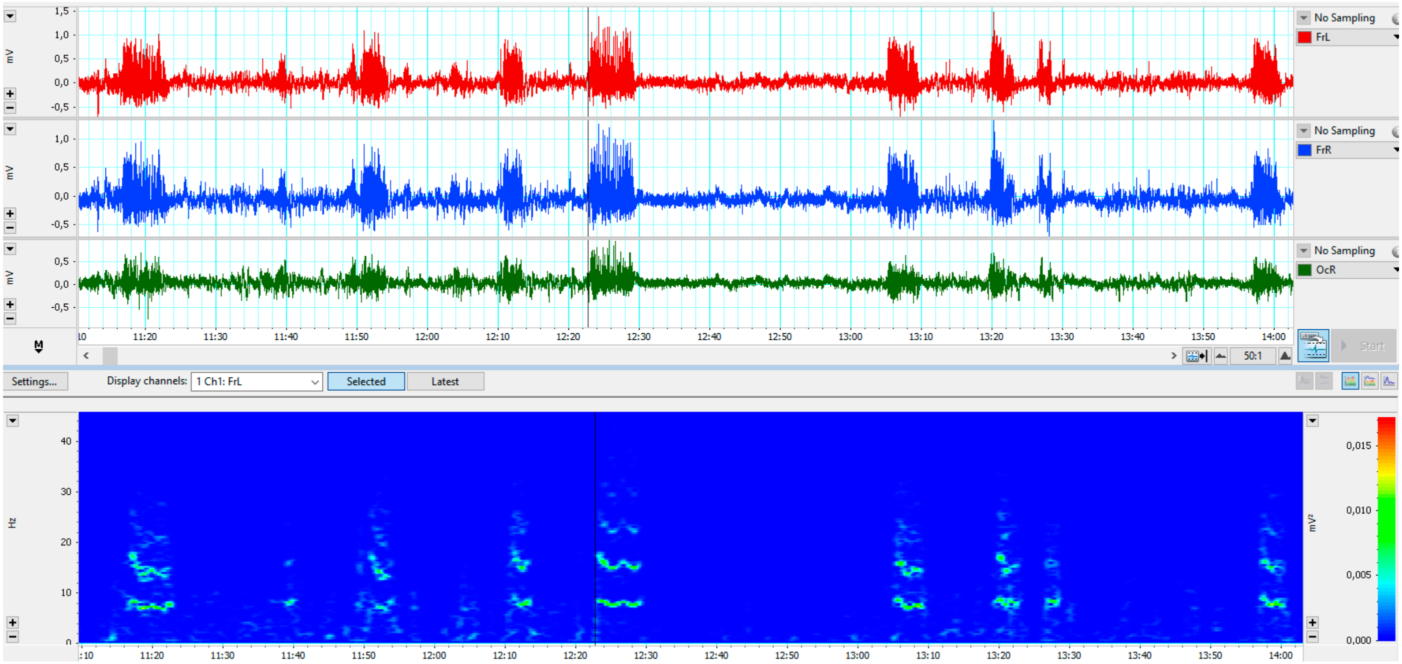

Figure S1.1. Spontaneous SWDs recorded in an 8-month-old female WAG/Rij rat and visualized in LabChart 8.0. Three-channel epidural EEG was recorded and abbreviated as FrL and FrR – frontal cortical left and right leads; OcR – occipital cortical lead. Time is given in mm:ss. The bottom graph shows an FFT power spectrum computed with 1024 size and 95,5 % window overlap.

The full length EEG was processed by continuous wavelet transform with the complex Morlet basic function. Wavelet power was computed in the main frequency band of SWDs, [8-10 Hz], and [17-20 Hz]. The threshold values of wavelet power for each band were chosen individually by testing different values and seeing which produced the most accurate results. SWDs were detected when wavelet power in both bands exceeded threshold values. To improve the method's selectivity, the seizure activity's minimal duration was set to 2 s.

Figure S1.2a shows the program's interface developed by the group of Prof. Alexander Hramov. We set frequency Ranges: [8-10] Hz and [17-20] Hz, minimal duration of SWD = 2000 ms and the percent of power decrease during SWDs that was necessary to correctly define the end of SWDs. Figure S1.2b shows the outcomes with SWDs detection results.

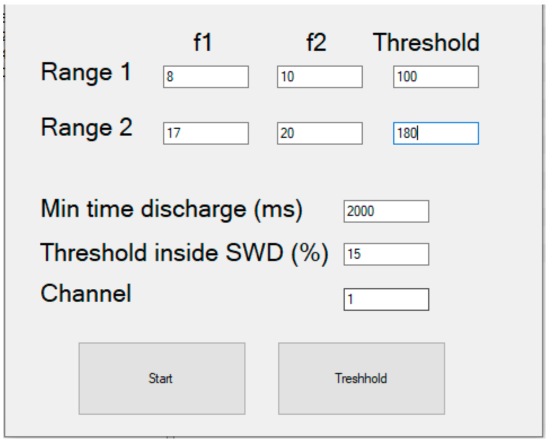

(*a*)

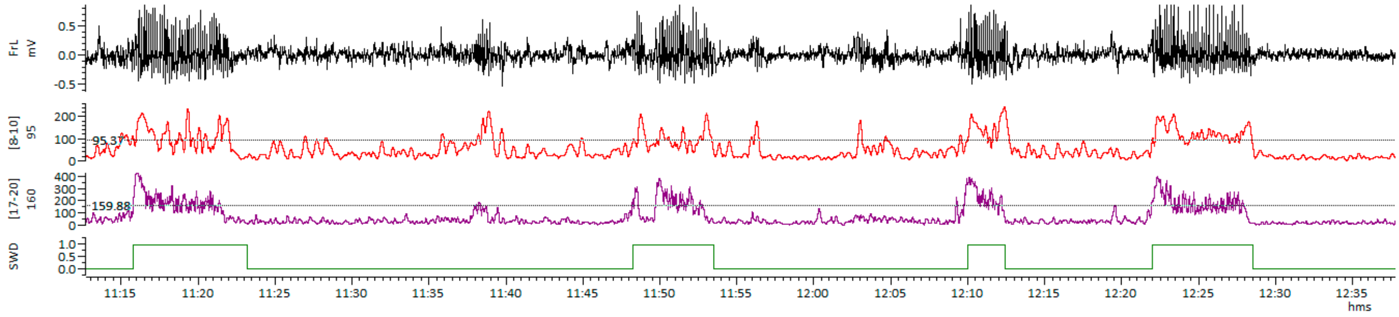

(*b*)

Figure S1.2. Wavelet-based automatic detection of SWDs. (***a***) Program interface with parameters for frequency Range 1 [8–10 Hz] and Range 2 [17–20 Hz], minimal duration of SWD = 2000 ms, threshold for wavelet power variability in % and EEG channel. (***b***) Results of the automatic detection of SWDs. The frontal left lead - the upper channel (black). The middle two channels show the wavelet power computed in the frequency band [8–10 Hz] and 1st harmonics - [17–20 Hz], where the threshold levels are indicated by horizontal lines. The bottom channel (green) shows the detections.

References

1. Coenen, A.M.L.; Van Luijtelaar, E.L.J.M. The WAG/Rij Rat Model for Absence Epilepsy: Age and Sex Factors. *Epilepsy Res.* **1987**, *1*, 297–301, doi:10.1016/0920-1211(87)90005-2.
2. van Luijtelaar, G.; van Oijen, G. Establishing Drug Effects on Electrocorticographic Activity in a Genetic Absence Epilepsy Model: Advances and Pitfalls. *Front. Pharmacol.* **2020**, *11*, 395, doi:10.3389/fphar.2020.00395.
