# Supplementary material for: Alpha2 Adrenergic Modulation of Spike-Wave Epilepsy: Experimental Study of Pro-Epileptic and Sedative Effects of Dexmedetomidine
Source: Int J Mol Sci. 2023 May 29;24(11):9445. doi: 10.3390/ijms24119445 (PMC10254047; doi:10.3390/ijms24119445)
Supplement: Supplementary file 1 [file ijms-24-09445-s001.zip › Supplementary File S2 - non-epileptics.pdf]

## Supplementary S2
## Spike-wave discharges in the control non-epileptic group (NEW rat substrain)

Spike-wave discharges were common in WAG/Rij subjects, but they were almost completely absent in non-epileptic control subjects (NEW). The male subject M2 displayed 13 SWD per hour in average (Table S2.1) showing epileptic phenotype. This subject was moved from the control non-epileptic to the epileptic group.

**Table S2.1.** Results of automatic detection of spike-wave discharges in non-epileptic control subjects (NEW) during baseline

| | Rats and recordings | | | | | SWDs | | | | |
|---|---|---|---|---|---|---|---|---|---|---|
| | ID | Date of birth | Date of recording | AGE, month s | Phase of cycle | Length of recogn., h | Total SWD's duration | SWD's density s/per hour | Mean SWD duration | Total number of SWDs |
| males | **M1** | 19.12.2021 | 25.05.2022 | 5.2 | | 19.56 | 2.3 | 0.12 | 2.3 | 1 |
| | **M2*** | 19.12.2021 | 18.07.2022 | 7 | | 11.11 | 428.1 | 38.53 | 2.93 | 146 |
| | **M3** | 19.12.2021 | 11.08.2022 | 7.8 | | 15.26 | 5.17 | 0.34 | 2.59 | 2 |
| | **M4** | 19.11.2021 | 11.08.2022 | 8.8 | | 24.85 | 2.8 | 0.11 | 2.8 | 1 |
| | **M5** | 19.11.2021 | 31.08.2022 | 9.5 | | 20.72 | 0 | 0 | - | 0 |
| | **M6** | 19.11.2021 | 31.10.2022 | 11.5 | | 21.72 | 0 | 0 | - | 0 |
| | | | | | | | | | | |
| females | **F1** | 19.11.2021 | 18.07.2022 | 8 | | 11.11 | 3.6 | 0.32 | 3.6 | 1 |
| | **F2** | 19.11.2021 | 09.08.2022 | 8.8 | proestrus | 20.11 | 2.3 | 0.11 | 2.3 | 1 |
| | **F3** | 19.12.2021 | 17.08.2022 | 8 | | 21.72 | 4.5 | 0.21 | 2.25 | 2 |
| | **F4** | 19.12.2021 | 31.08.2022 | 8.5 | diestrus | 20.72 | 13 | 0.63 | 2.6 | 5 |
| | **F5#** | 19.11.2021 | 15.08.2022 | 9 | diestrus | 21.72 | 0 | 0 | - | 0 |
| | **F6#** | 13.11.2021 | 09.08.2022 | 9 | proestrus | 20.11 | 0 | 0 | - | 0 |
| | **F7** | 13.11.2021 | 15.08.2022 | 9.2 | proestrus | 21.72 | 6.5 | 0.3 | 3.25 | 2 |
| | **F8** | 13.11.2021 | 17.08.2022 | 9.2 | | 21.72 | 0 | 0 | - | 0 |
| | **F8** | 13.11.2021 | 31.10.2022 | 11.7 | | 21.72 | 3.4 | 0.16 | 3.4 | 1 |

* - Subject M2 was excluded from analysis, because it has an epileptic phenotype
\# - Subjects F5 and F6 had non-epilpetic phenotype in baseline and showed SWDs after i.p. injection of Dexmedetomidine

Dexmedetomidine (Dex) in doses ranging from 0.004 to 0.034 mg/kg was intraperitoneally injected in NEW rats (Figure S2.1).

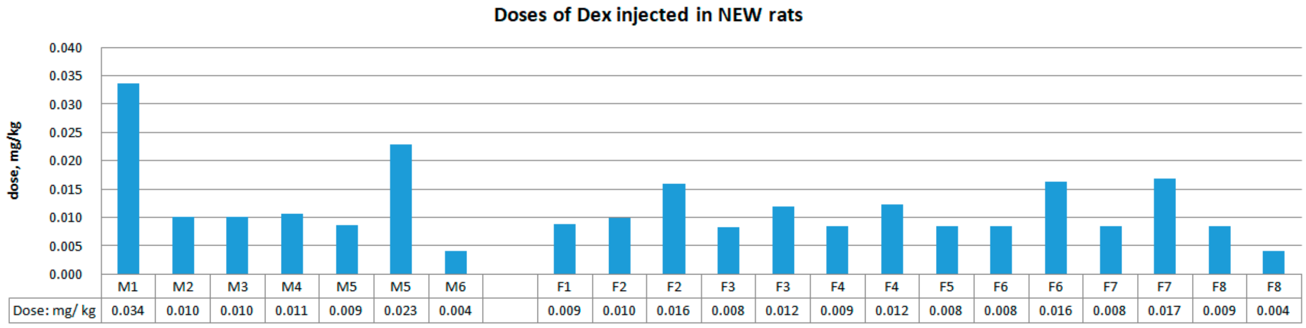


**Figure S2.1.** Doses of Dexmedetomidine (Dex) injected in the group of NEW rats.

Two female subjects (F5 and F6) with the non-epileptic phenotype determined in baseline (Table S1) showed SWDs after administration of Dexmedetomidine in dose 0.008 mg/kg (both subjects). In the rest 11 control NEW rats, Dexmedetomidine did not provoke genuine SWDs. The results of the automatic SWDs detection are shown in Table S2.2.

**Table S2.2.** Results of automatic detection of spike-wave discharges in non-epileptic control subjects (NEW) after i.p. administration of Dexmedetomidine.

| | Rats and recordings | | | | | SWDs | | | | |
|---|---|---|---|---|---|---|---|---|---|---|
| | ID | Date of recording | AGE, months | Phase of cycle | Dex. dose, mg/kg | Length of recogn., h | Length of recogn., h | SWD density s/per hour | Mean SWD duration | nSWD |
| | M1 | 26.05.2022 | 5.3 | | 0.034 | 10.66 | 0 | 0 | - | 0 |
| | M2 | 19.07.2022 | 7.1 | | 0.010 | 6.22 | 536 | 86.14 | 3.01 | 178 |
| | M3 | 12.08.2022 | 7.9 | | 0.010 | 20.72 | 2.8 | 0.1 | 2.08 | 1 |
| males | M4 | 12.08.2022 | 8.9 | | 0.011 | 20.72 | 0 | 0 | - | 0 |
| | M5 | 01.09.2022 | 9.5 | | 0.009 | 23.56 | 2.2 | 0.09 | 2.2 | 1 |
| | M5 | 14.09.2022 | 10 | | 0.023 | 21.72 | 0 | 0 | - | 0 |
| | M6 | 01.11.2022 | 11.5 | | 0.004 | 23.56 | 4.9 | 0.21 | 2.45 | 2 |
| | | | | | | | | | | |
| | F1 | 19.07.2022 | 8.1 | | 0.009 | 6.22 | 0 | 0 | - | 0 |
| | F2 | 10.08.2022 | 8.8 | proestrus | 0.010 | 22.22 | 15 | 0.68 | 2.5 | 6 |
| | F2 | 12.09.2022 | 9.9 | diestrus | 0.016 | 22.78 | 0 | 0 | - | 0 |
| | F3 | 18.08.2022 | 8.1 | | 0.008 | 22.78 | 0 | 0 | - | 0 |
| | F3 | 13.09.2022 | 8.9 | proestrus | 0.012 | 23.56 | 0 | 0 | - | 0 |
| | F4 | 01.09.2022 | 8.5 | proestrus | 0.009 | 23.56 | 3.5 | 0.15 | 3.5 | 1 |
| | F4 | 13.09.2022 | 8.9 | diestrus | 0.012 | 23.56 | 0 | 0 | - | 0 |
| females | F5 | 16.08.2022 | 9 | proestrus | 0.008 | 21.72 | 430 | 19.8 | 6.14 | 70 |
| | F6 | 10.08.2022 | 9 | diestrus | 0.008 | 22.22 | 42.8 | 1.93 | 2.38 | 18 |
| | F6 | 12.09.2022 | 9.9 | diestrus | 0.016 | | | | | |
| | F7 | 16.08.2022 | 9.2 | proestrus | 0.008 | 22.39 | 0 | 0 | - | 0 |
| | F7 | 14.09.2022 | 10.2 | proestrus | 0.017 | 21.72 | 9.0 | | | 3 |
| | F8 | 18.08.2022 | 9.3 | | 0.004 | 23.56 | 0 | 0 | - | 0 |
| | F8 | 01.11.2022 | 11.7 | | 0.009 | 22.78 | 12.5 | 0.55 | 2.5 | 5 |

In non-epileptic subjects, i.p. injections of Dex in low doses (0.004-0.034 mg/kg) did not provoke genuine SWDs. Figure S2.2a demonstrates that the power at the central frequency of 8-10 Hz was not high enough to reach the threshold level, therefore, no genuine SWDs were detected. However, injections of Dex elicited epileptiform activity in the form of irregular spikes, 4-8 Hz sharp waves and occasional spike-wave complexes (Figure S2.2*b*,*c*). This epileptiform activity did not meet the criteria for SWDs. Four-8 Hz oscillations were defined as the rudimentary SWDs in the frequency band of 4-8 Hz (rudSWDs, Figure S2.2*c*).

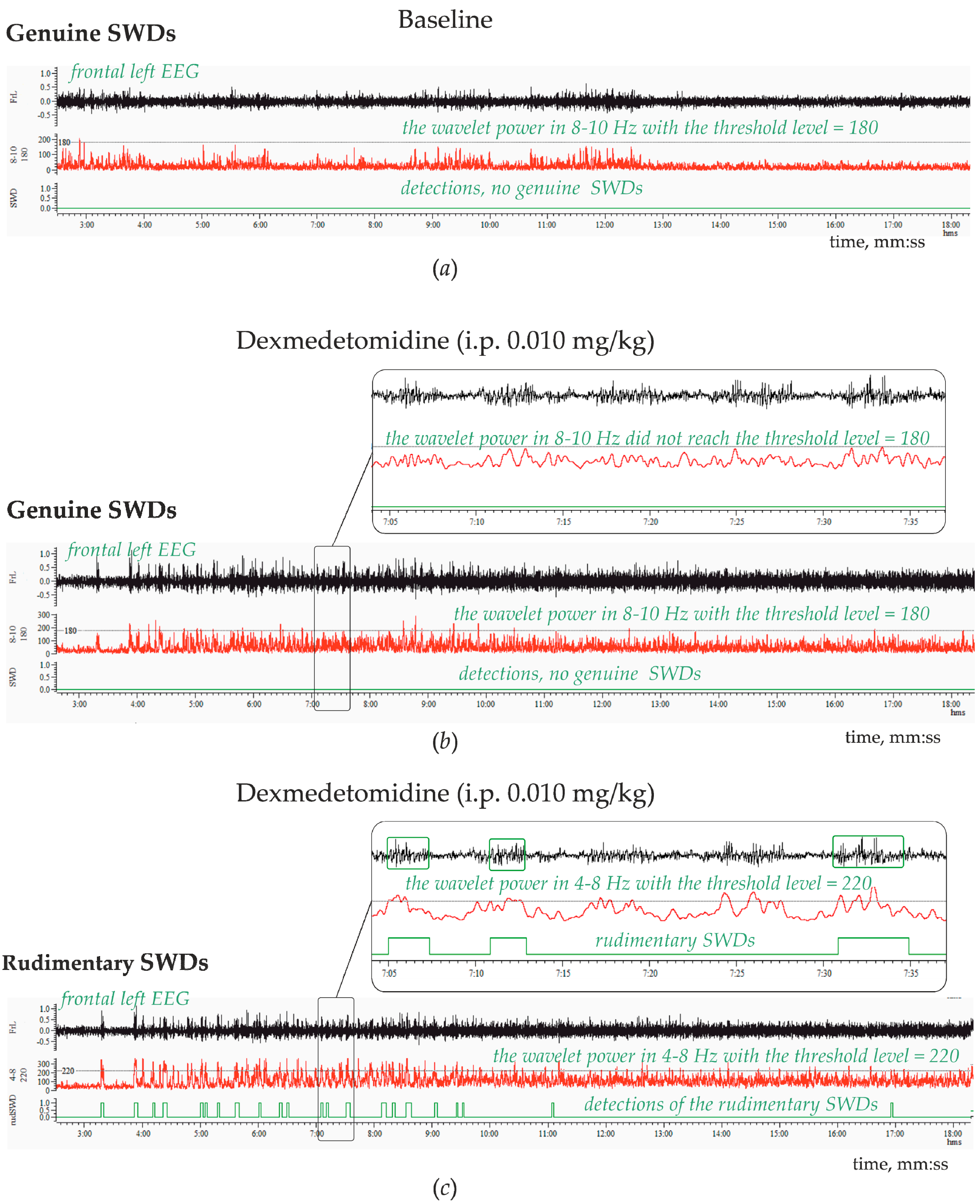


**Figure S2.2.** Results of SWDs detection in non-epileptic rat. Raw EEGs are shown in black; wavelet power in red; SWD detections (binary responses) as green lines. (***a***) In baseline, wavelet power at 8-10 Hz did not reach the threshold level, therefore no genuine SWDs. (***b***) After injection of Dex, wavelet power at 8-10 Hz did not reach the threshold level, and genuine SWDs were not detected. (***c***) After the injection of Dex, the rudimentary SWDs were detected based on the elevation of the wavelet power at 4-8 Hz, which is a lower frequency than in genuine SWDs.
