# Supplementary material for: Alpha2 Adrenergic Modulation of Spike-Wave Epilepsy: Experimental Study of Pro-Epileptic and Sedative Effects of Dexmedetomidine
Source: Int J Mol Sci. 2023 May 29;24(11):9445. doi: 10.3390/ijms24119445 (PMC10254047; doi:10.3390/ijms24119445)
Supplement: Supplementary file 1 [file ijms-24-09445-s001.zip › Supplementary File S3 - epileptics.pdf]

**Supplementary File S3**
**Individual results of the automatic analysis of SWDs**

**Table S3.1.** Information about rat subjects used in the control and the experimental groups

| Strain | N of subjects | Sex | Body weight, g | IDs |
|---|---|---|---|---|
| Non-epileptic, control | | | | |
| NEW | 5 | males | 355-446 | M1, M3-M6 |
| | 8 | females | 230-253 | F1-F8 |
| Total | 13 | | | |
| Epileptic phenotype (spontaneous SWDs in baseline) | | | | |
| WAG/Rij | 2 | males | 356-367 | W11, W12 |
| | 9 | females | 226-357 | W1-W8, W10 |
| Wistar | 1 | female | 273-275 | W9 |
| NEW | 1 | male | 400 | M2 |
| Total | 13 | | | |

*- Non-epileptic subjects F4 and F5 exhibited the genuine SWDs after Dex injections (the latent form of epilepsy).

**Table S3.2.** Results of cluster analysis of the epileptic group (13 rats)

| Cluster 1 Low/no effect | | Cluster 2 Pro-epileptic effect | | Cluster 3 Severe absence (Epi-status, early) | | Cluster 4 Severe absence (Epi-status, late) | |
|---|---|---|---|---|---|---|---|
| ID | Dex dose | ID | Dex dose | ID | Dex dose | ID | Dex dose |
| W1 | 0.049 | W1 | 0.007 | W3 | 0.011 | W6 | 0.004 |
| W1 | 0.004 | W2 | 0.004 | W5 | 0.012 | W6 | 0.008 |
| W7 | 0.007 | W4 | 0.008 | W5 | 0.007 | | |
| W7 | 0.011 | W5 | 0.003 | | | | |
| W7 | 0.012 | W7 | 0.037 | | | | |
| W7 | 0.003 | W9 | 0.011 | | | | |
| W8 | 0.008 | W10 | 0.012 | | | | |
| W11 | 0.014 | W6 | 0.004 | | | | |
| W12 | 0.014 | W2 | 0.008 | | | | |
| W3 | 0.003 | | | | | | |
| M2 | 0.010 | | | | | | |
| Average | | | | | | | |

The following plots demonstrate density of SWDs after dexmedetomidine injections in different doses and during baseline. Density of SWDs was computed in s with 5 min bin

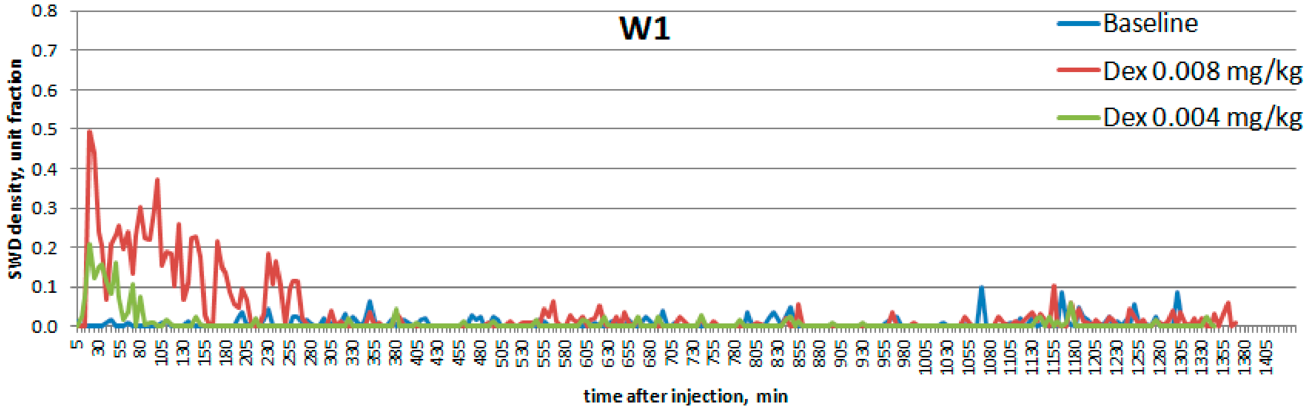


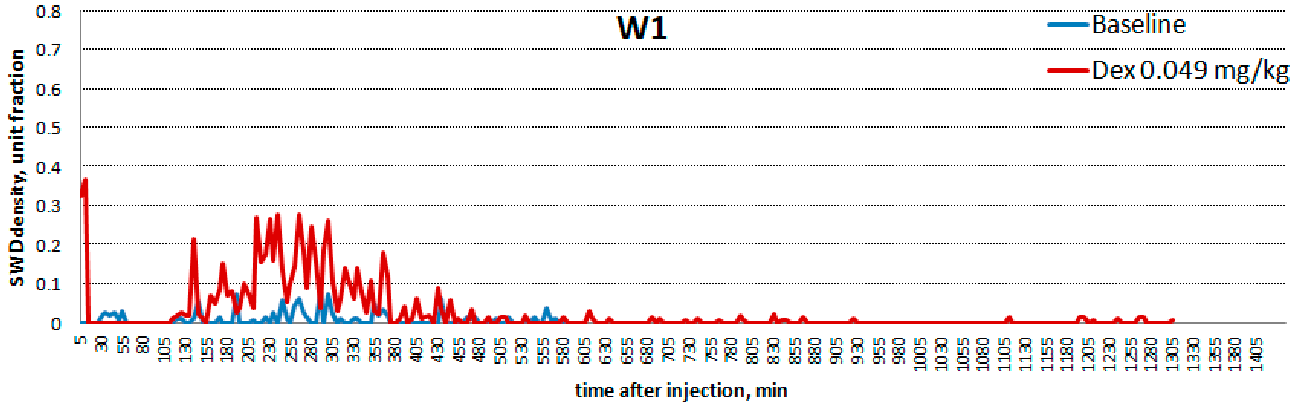


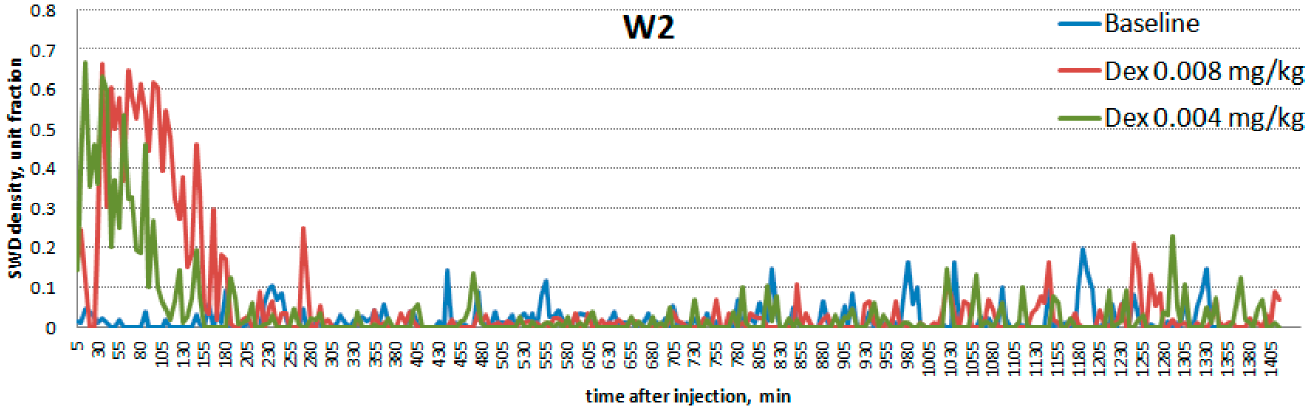


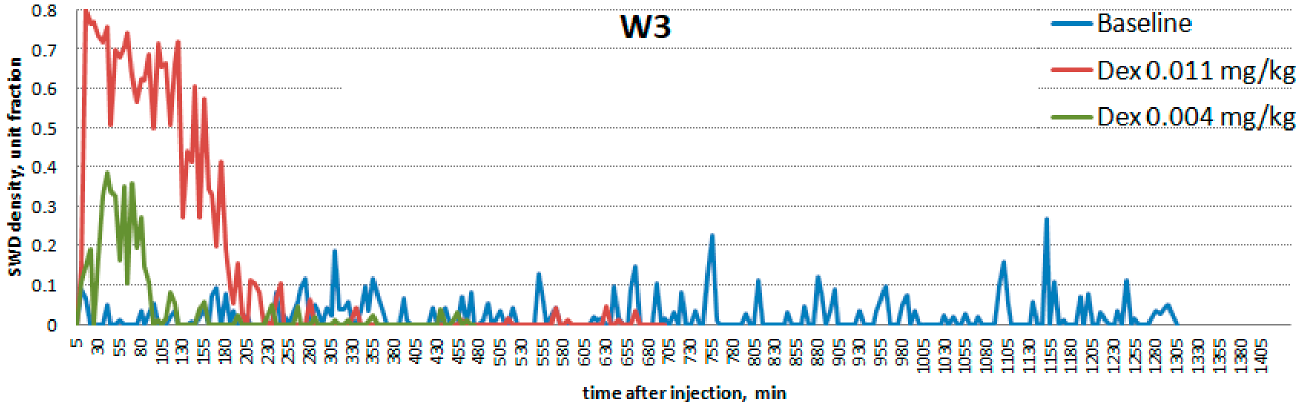

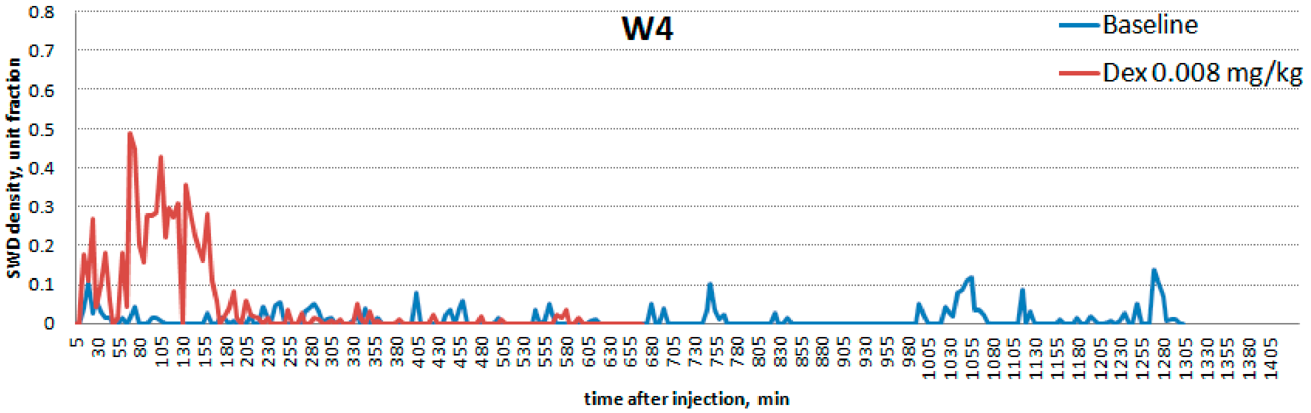
W4
Baseline
Dex 0.008 mg/kg
SWD density, unit fraction
time after injection, min

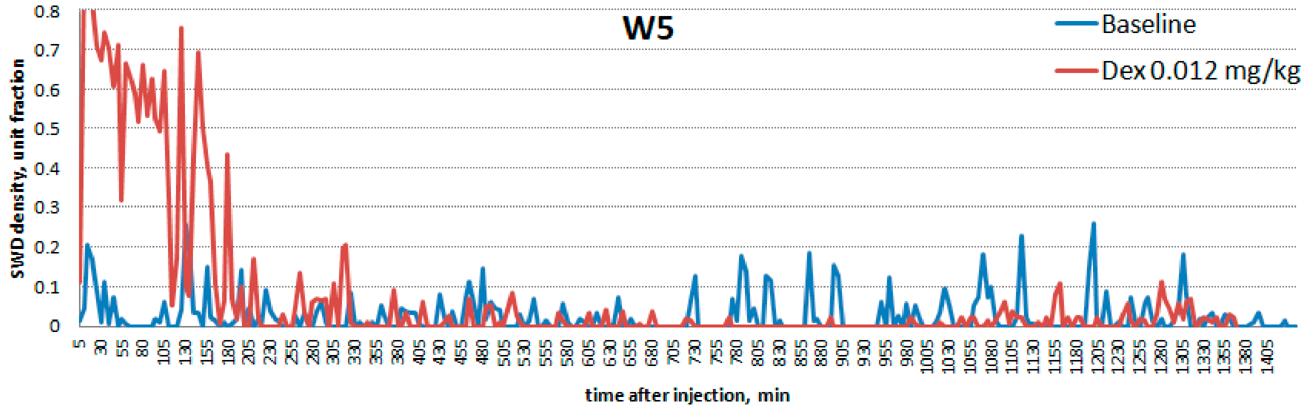
W5
Baseline
Dex 0.012 mg/kg
SWD density, unit fraction
time after injection, min

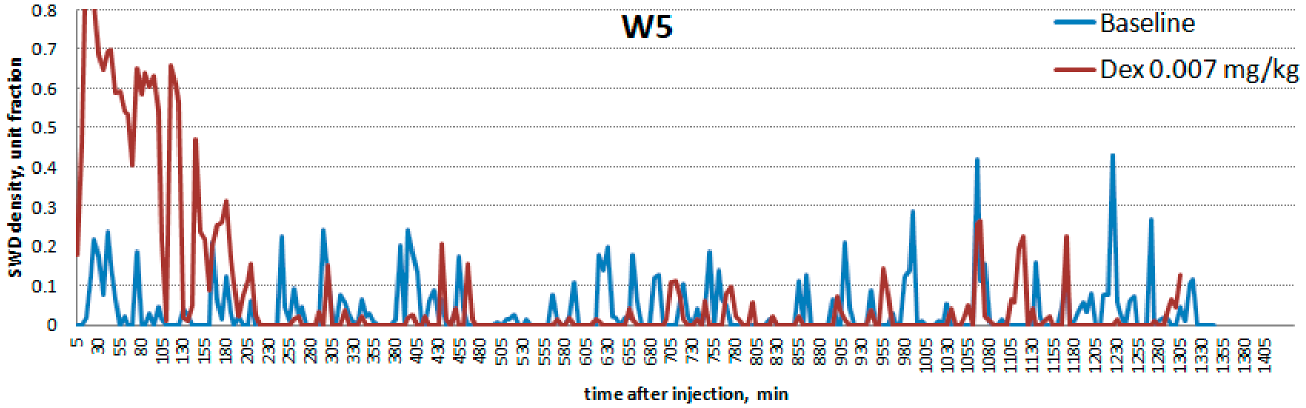
W5
Baseline
Dex 0.007 mg/kg
SWD density, unit fraction
time after injection, min

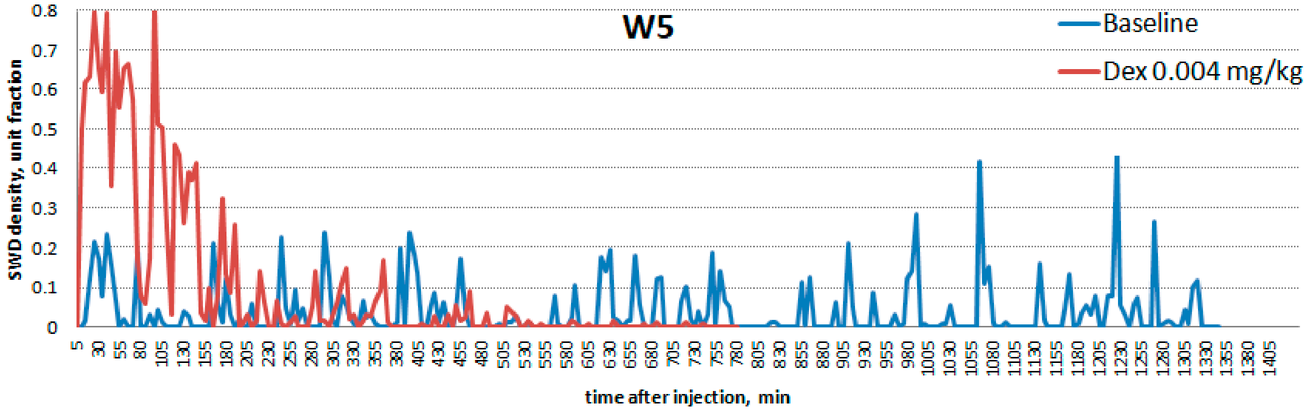
W5
Baseline
Dex 0.004 mg/kg
SWD density, unit fraction
time after injection, min

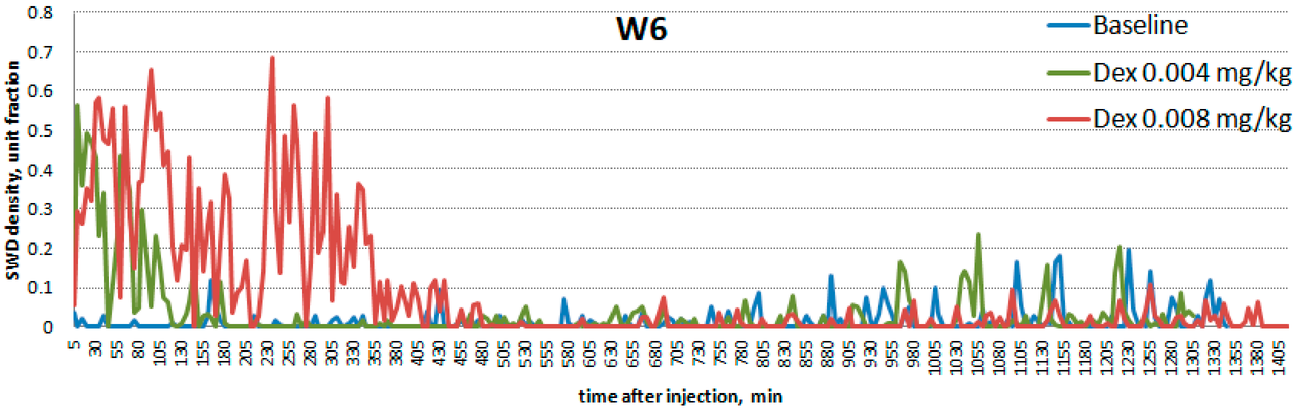
W6
Baseline
Dex 0.004 mg/kg
Dex 0.008 mg/kg
SWD density, unit fraction
time after injection, min

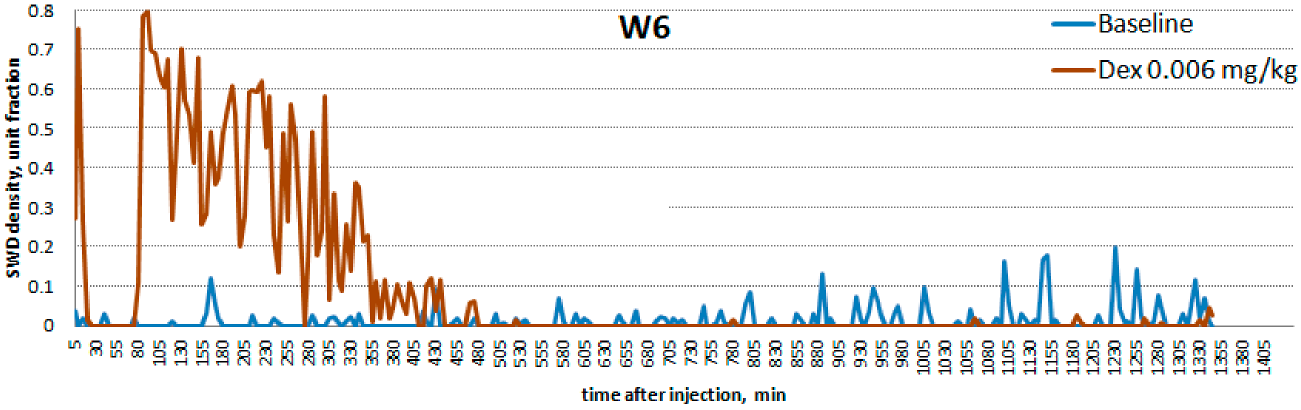
W6
Baseline
Dex 0.006 mg/kg
SWD density, unit fraction
time after injection, min

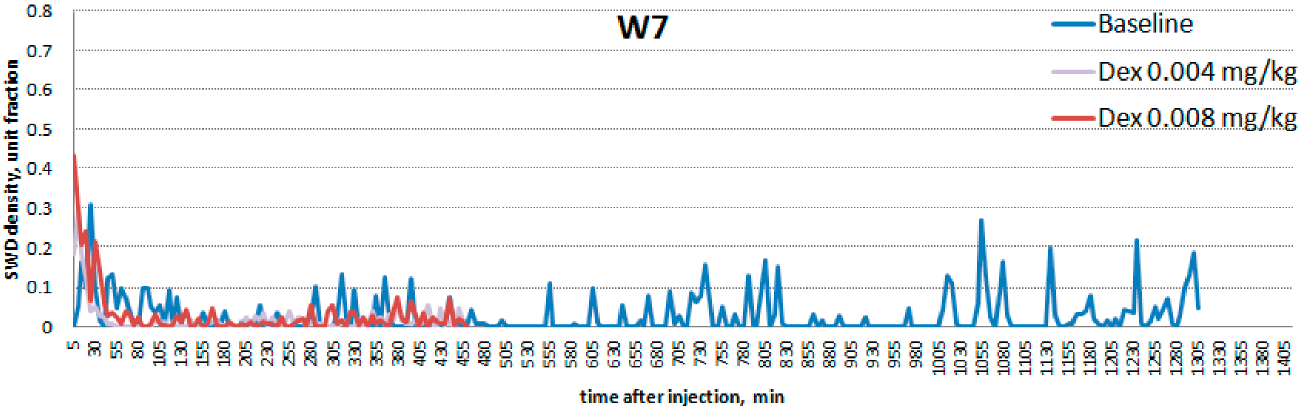
W7
Baseline
Dex 0.004 mg/kg
Dex 0.008 mg/kg
SWD density, unit fraction
time after injection, min

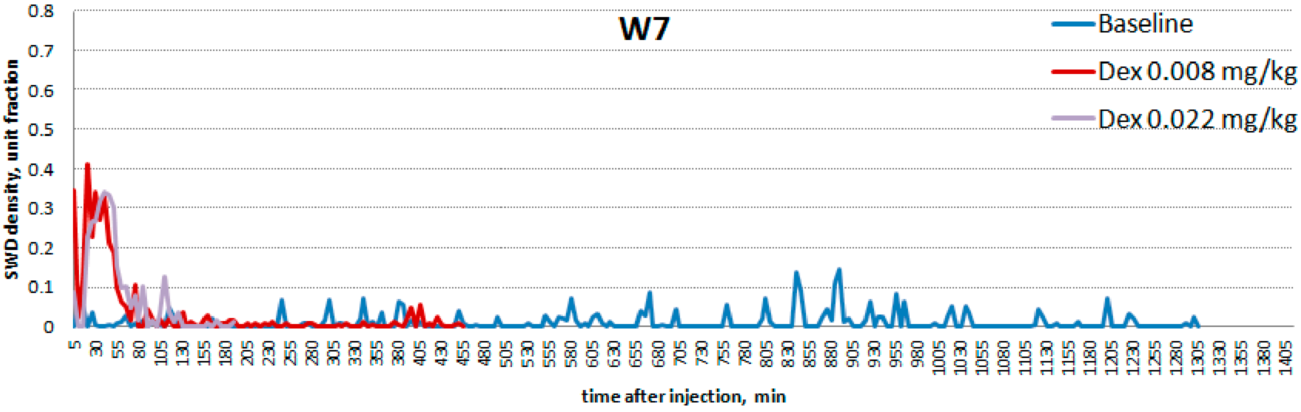
W7
Baseline
Dex 0.008 mg/kg
Dex 0.022 mg/kg
SWD density, unit fraction
time after injection, min

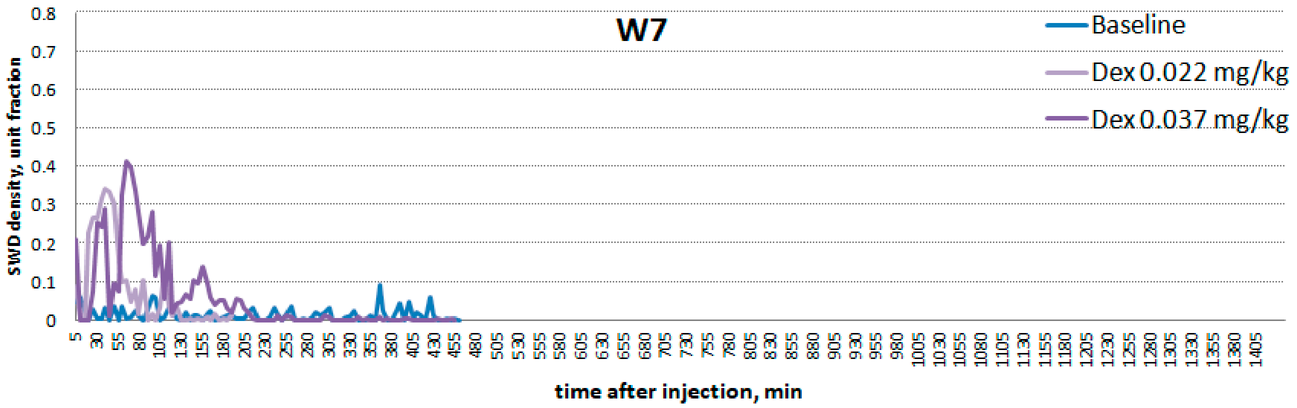
W7
Baseline
Dex 0.022 mg/kg
Dex 0.037 mg/kg
SWD density, unit fraction
time after injection, min
0
0.1
0.2
0.3
0.4
0.5
0.6
0.7
0.8

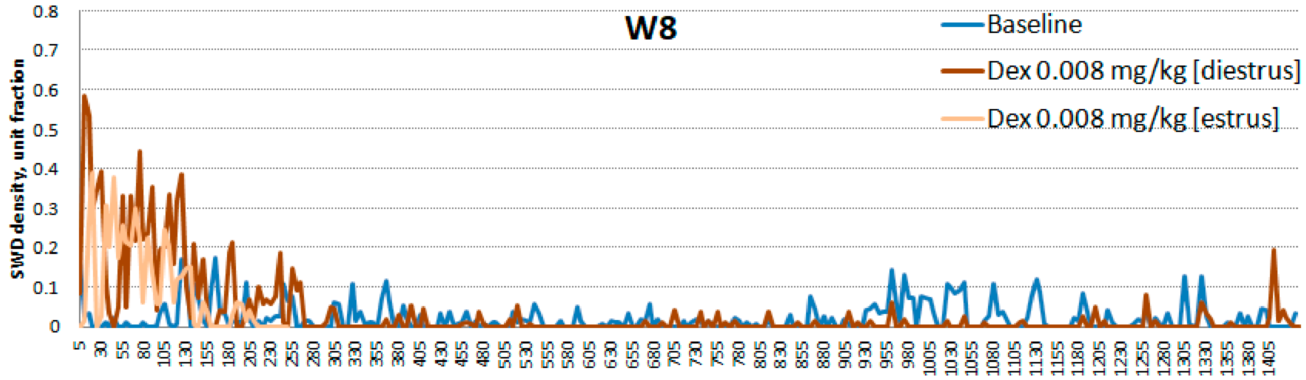
W8
Baseline
Dex 0.008 mg/kg [diestrus]
Dex 0.008 mg/kg [estrus]
SWD density, unit fraction
0
0.1
0.2
0.3
0.4
0.5
0.6
0.7
0.8

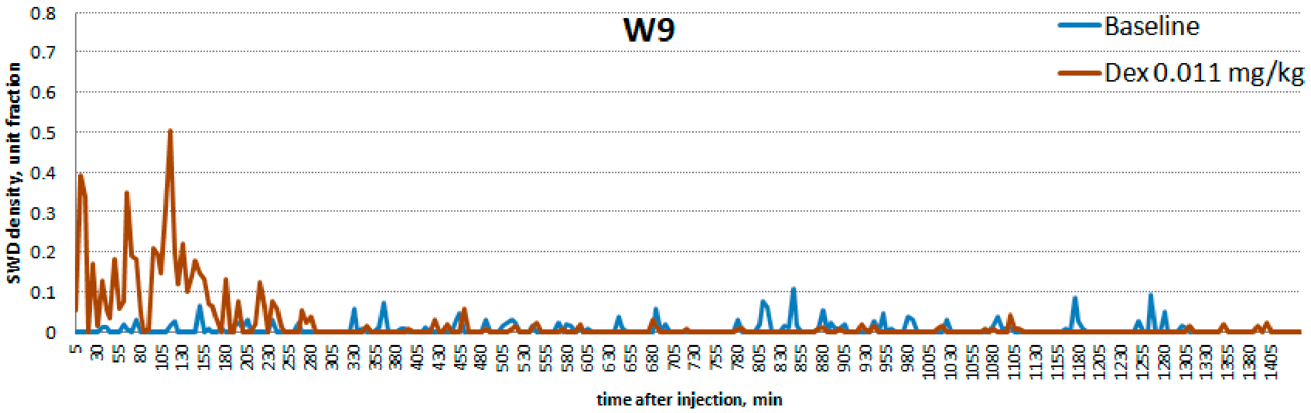
W9
Baseline
Dex 0.011 mg/kg
SWD density, unit fraction
time after injection, min
0
0.1
0.2
0.3
0.4
0.5
0.6
0.7
0.8

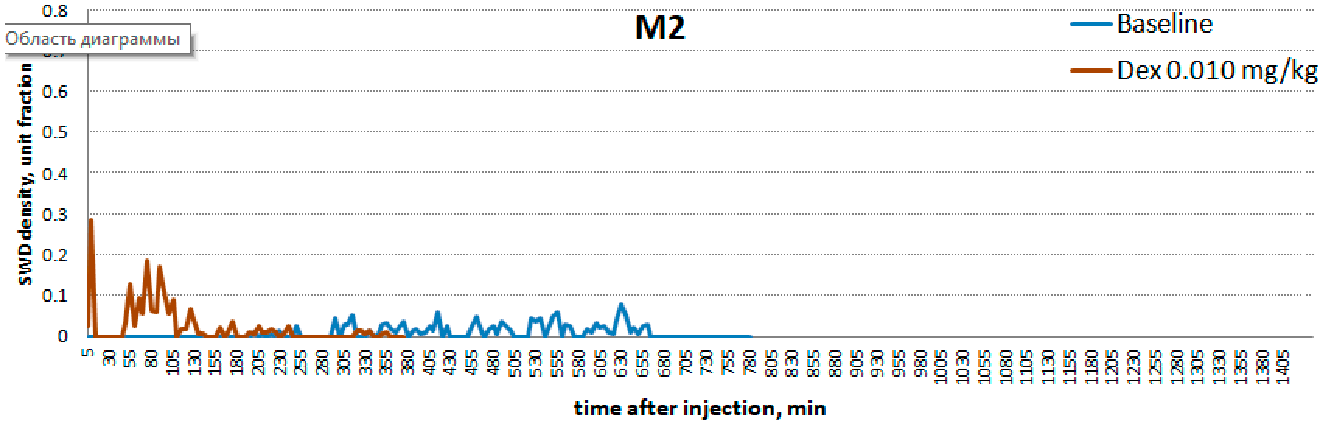
M2
Baseline
Dex 0.010 mg/kg
Область диаграммы
SWD density, unit fraction
time after injection, min
0
0.1
0.2
0.3
0.4
0.5
0.6
0.8

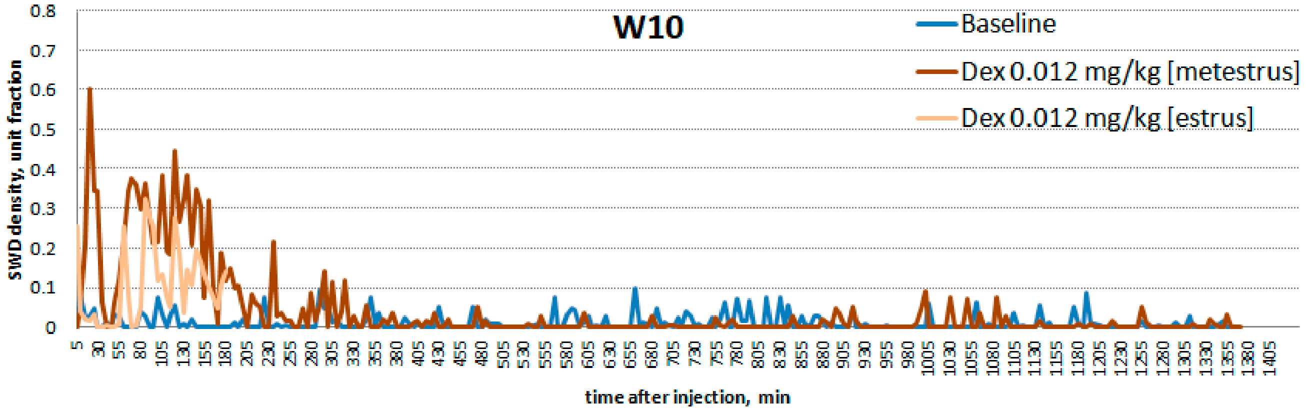
W10
Baseline
Dex 0.012 mg/kg [metestrus]
Dex 0.012 mg/kg [estrus]
SWD density, unit fraction
time after injection, min

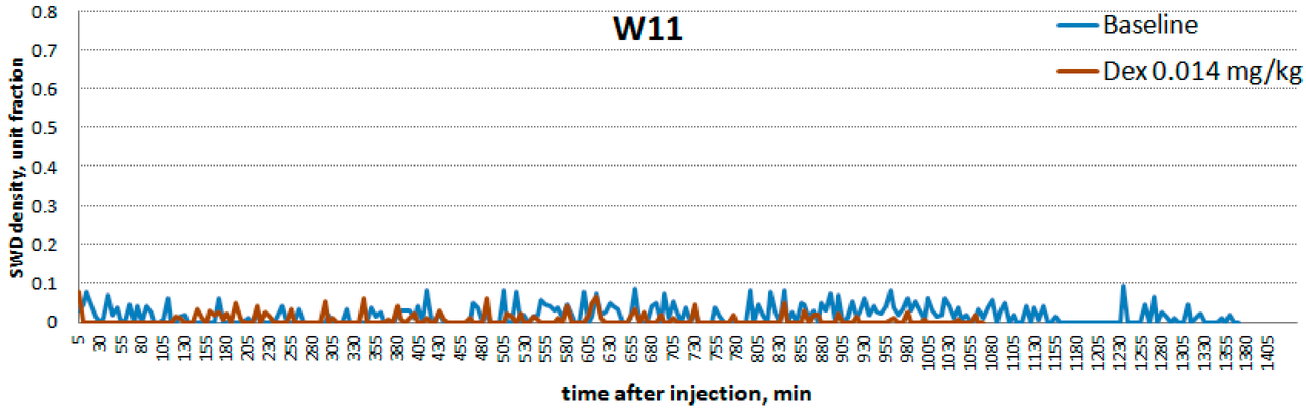
W11
Baseline
Dex 0.014 mg/kg
SWD density, unit fraction
time after injection, min

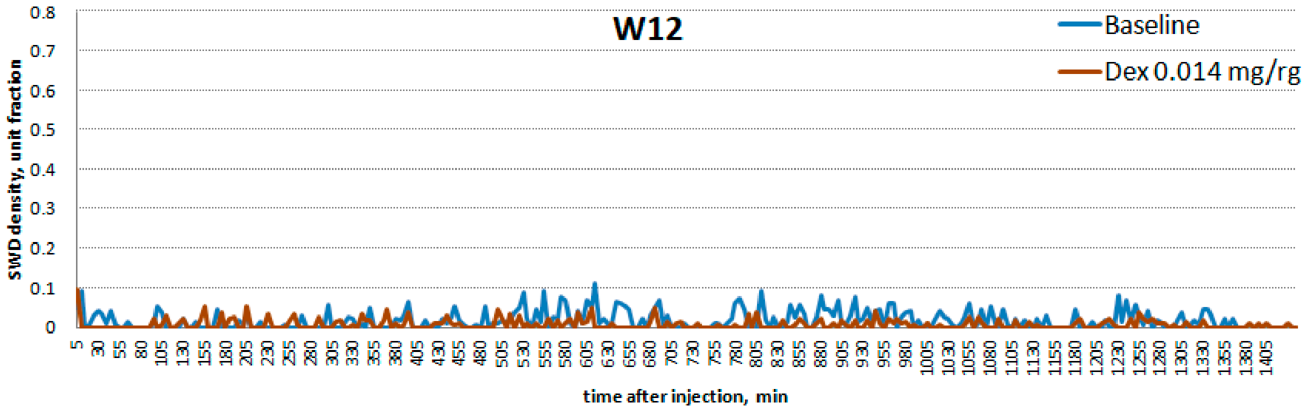
W12
Baseline
Dex 0.014 mg/rg
SWD density, unit fraction
time after injection, min

## Part 2. Medetomidine injections

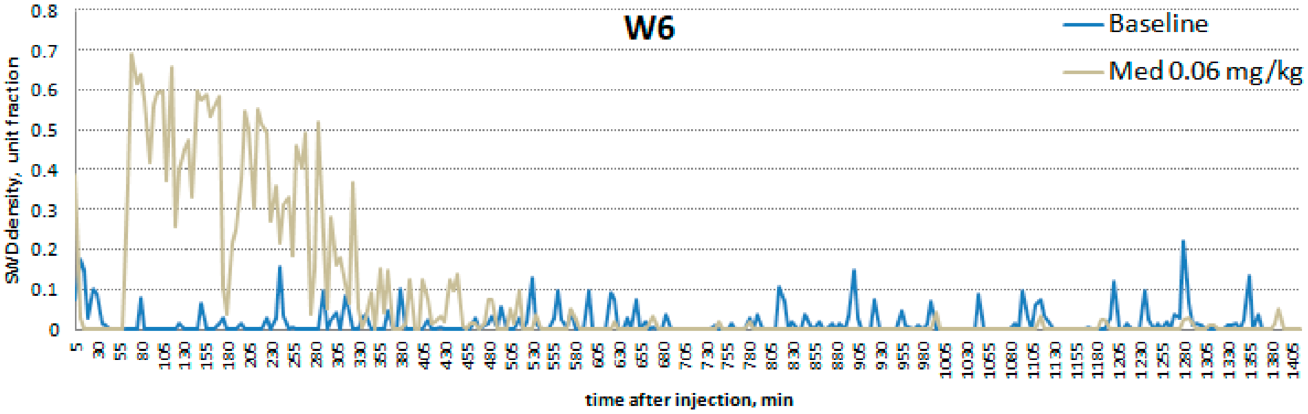


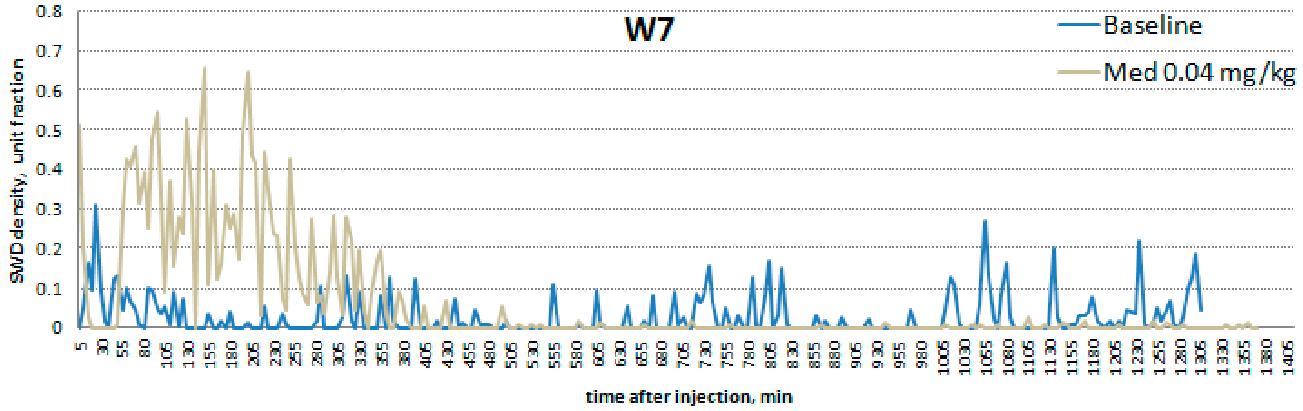


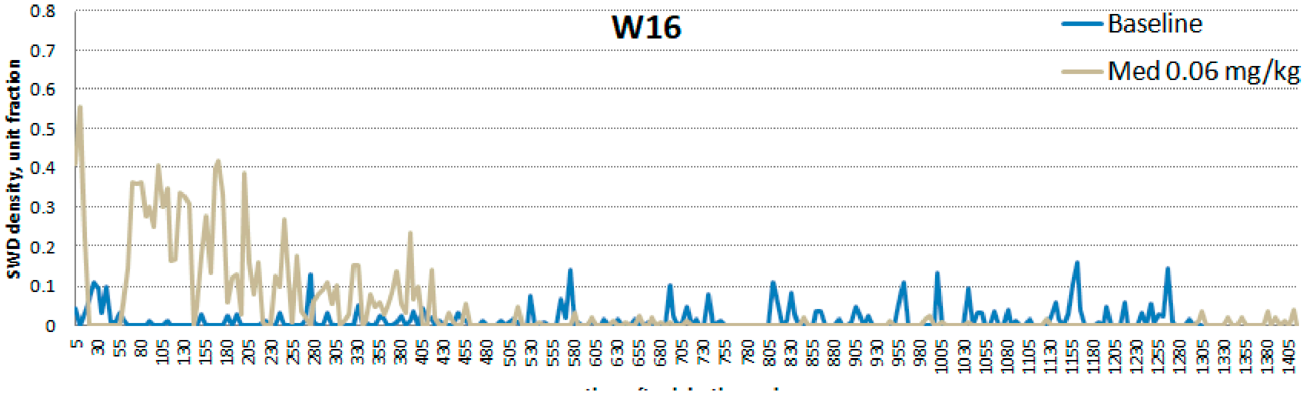

## Part 3. Xylasine injections

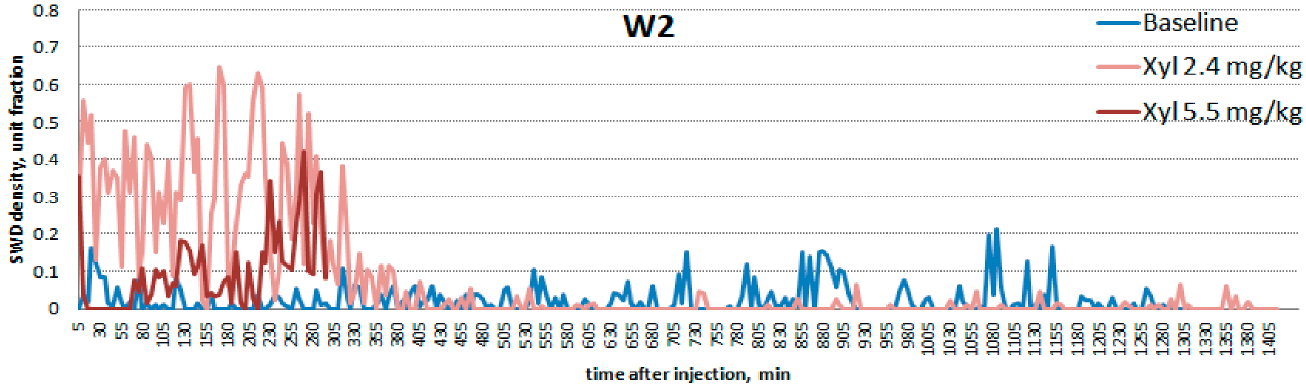


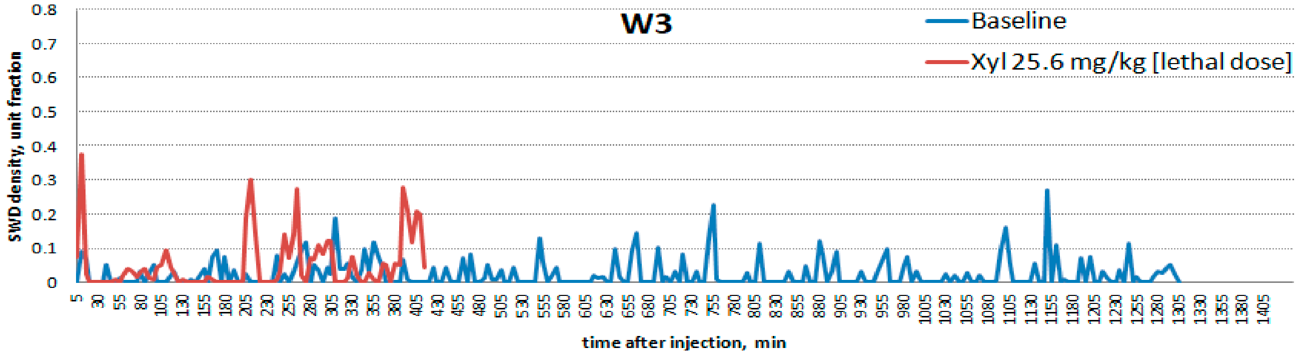


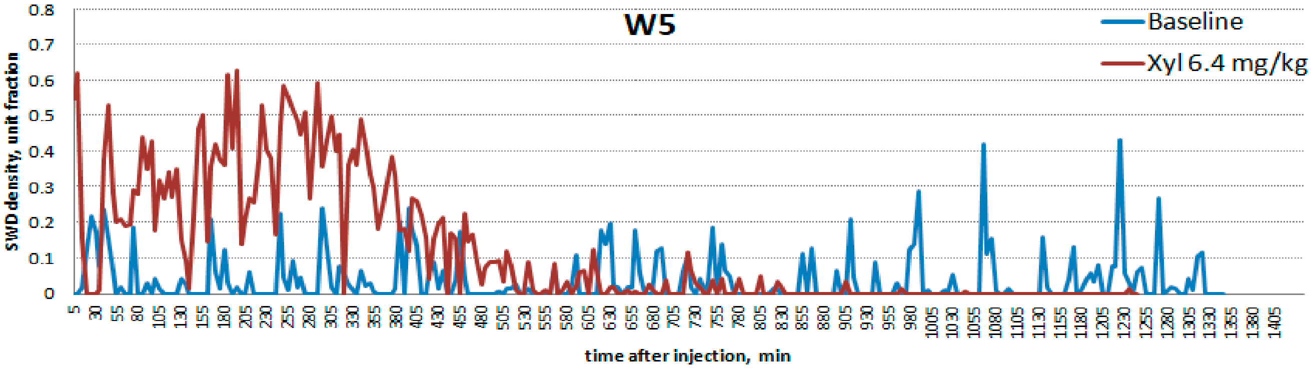


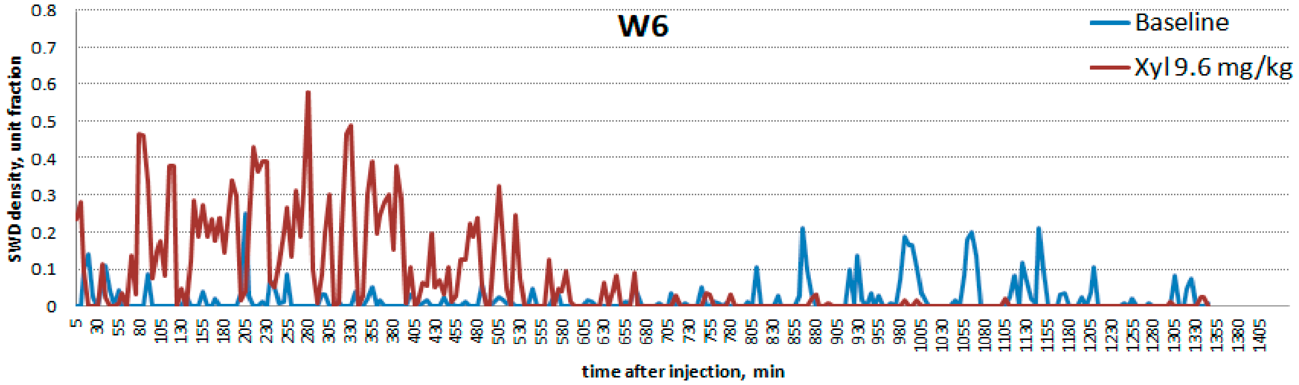


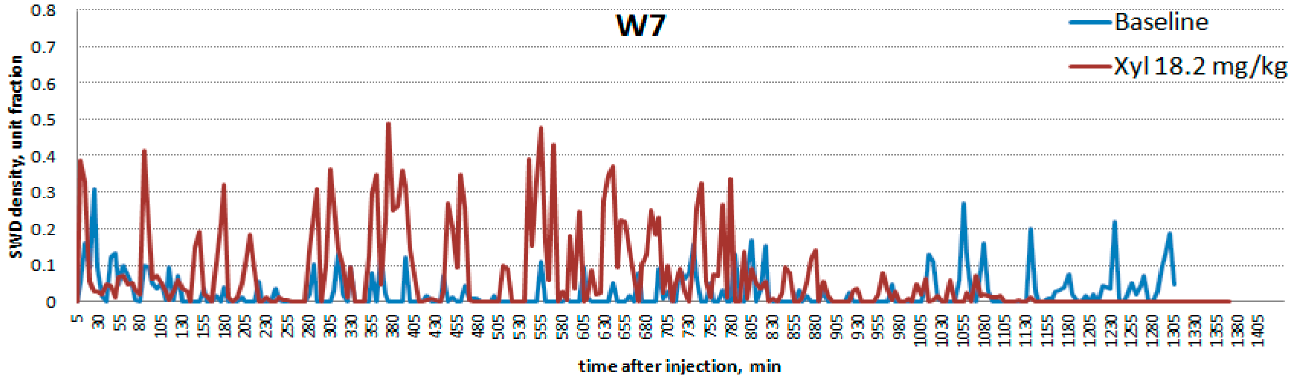

Part 4. Terazazine administration (per os)

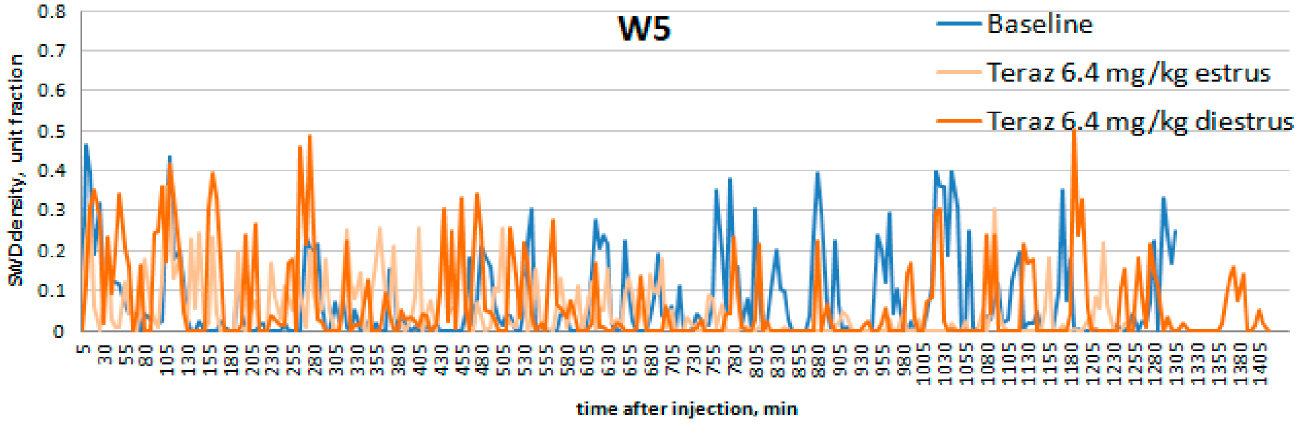


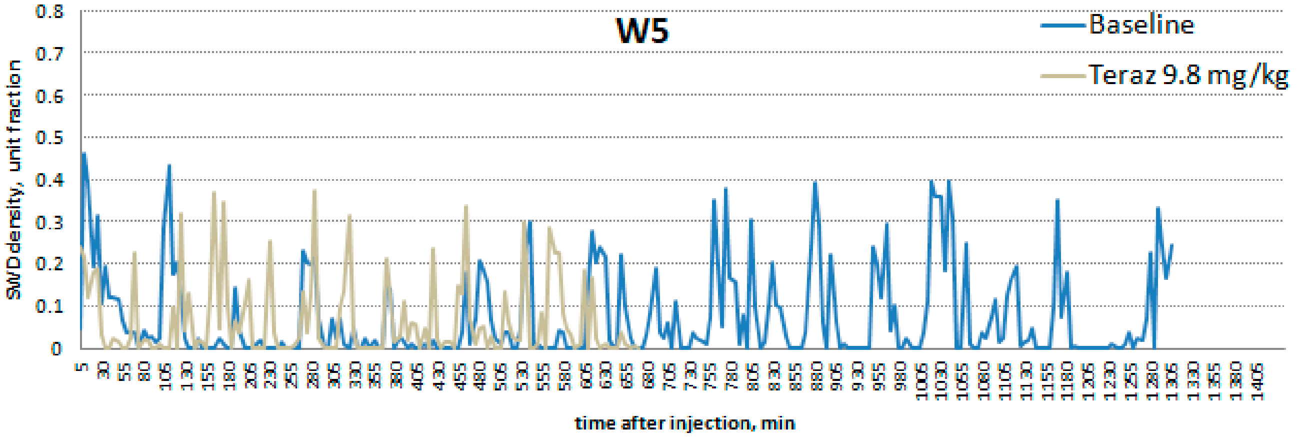


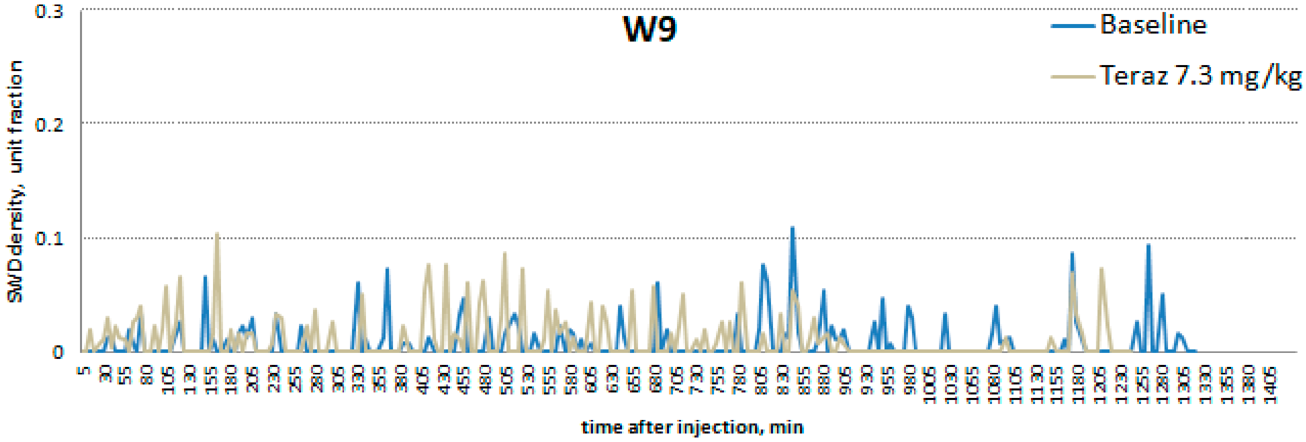


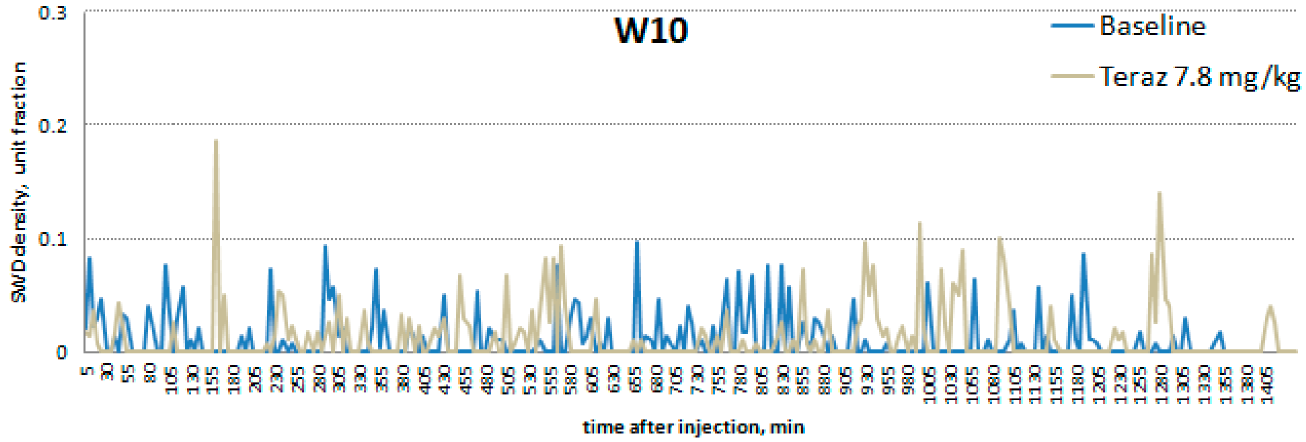

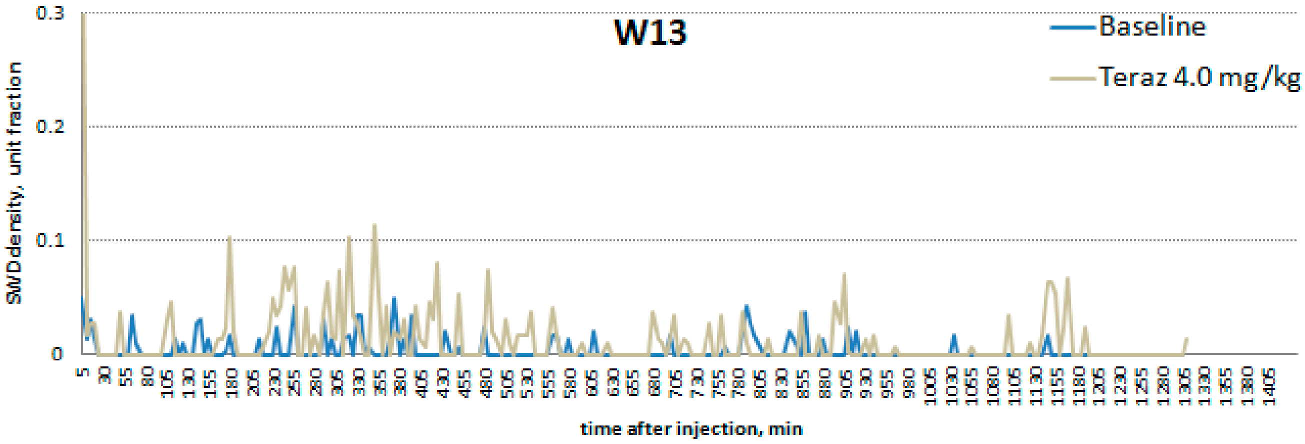
W13
Baseline
Teraz 4.0 mg/kg
SWD density, unit fraction
time after injection, min

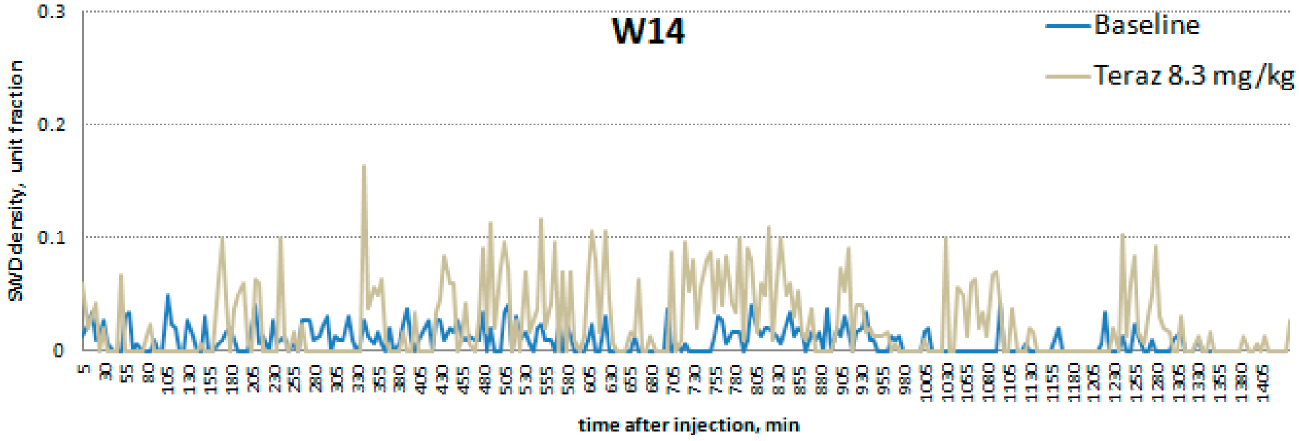
W14
Baseline
Teraz 8.3 mg/kg
SWD density, unit fraction
time after injection, min
