# Supplementary material for: Alpha2 Adrenergic Modulation of Spike-Wave Epilepsy: Experimental Study of Pro-Epileptic and Sedative Effects of Dexmedetomidine
Source: Int J Mol Sci. 2023 May 29;24(11):9445. doi: 10.3390/ijms24119445 (PMC10254047; doi:10.3390/ijms24119445)
Supplement: Supplementary file 1 [file ijms-24-09445-s001.zip › Supplementary File S4 - states.pdf]

Supplementary S4
EEG-based visual detection of behavioral states in rats after i.p. injection of Dexmedetomidine

Five states were identified based visual analysis of raw EEG, EEG power-spectrum and video records (when available): 1) wakefulness, 2) slow-wave sleep, 3) spike-wave activity, 4) deep sedation phase and 5) the state with mixed EEG patterns.

1. *Wakefulness* (Figure S4.1). This state was associated with body movements (large or small amplitude) or with still posture while preparing to fall asleep. The frontal left EEG during wakefulness showed low-amplitude (around 300 uV) fast activity. The power-density spectrum had two dominating frequency bands: delta (0.5–4 Hz) and theta (around 7 Hz) Wakefulness also included feeding periods (active wakefulness) when specific artifacts were present in the EEG.

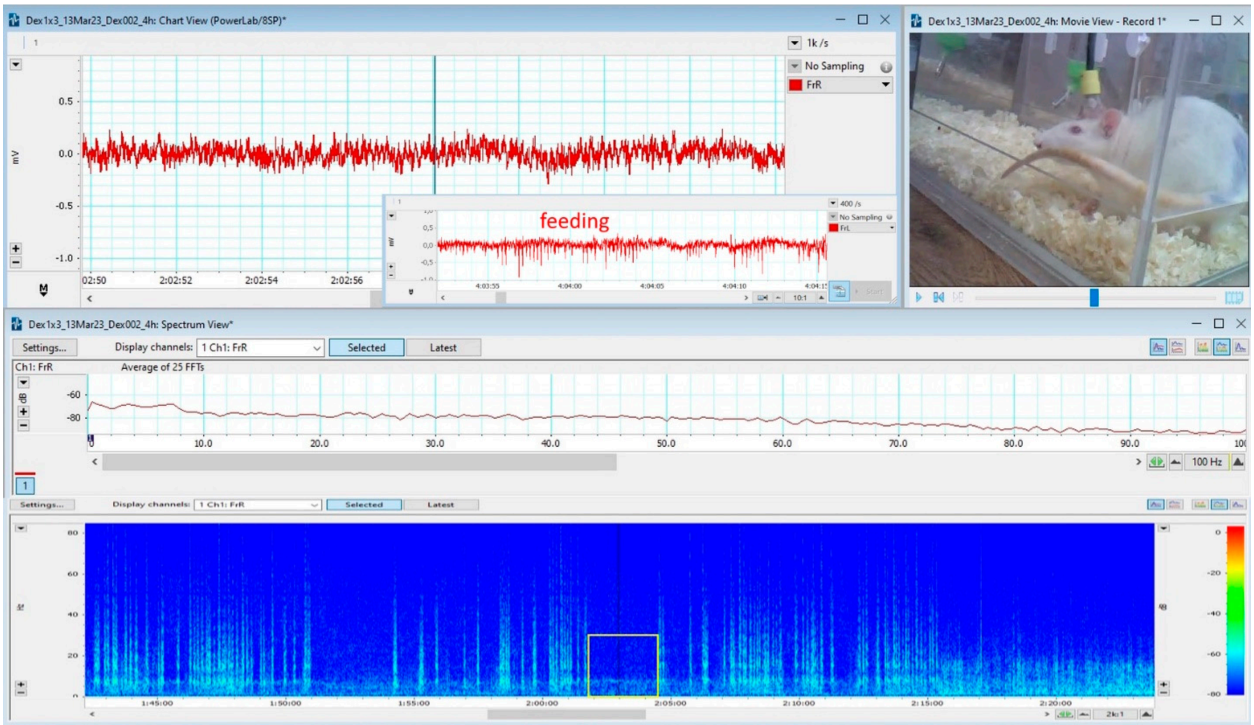

Figure S4.1. Wakefulness. The upper panel shows frontal left EEG with vertical blue line corresponding to video screenshot on the right, the inset shows typical feeding EEG artefacts. The middle panel shows power-spectrum of frontal left EEG during wakefulness period: delta (0.5-4 Hz) and theta (around 7 Hz) bands are dominated in the power spectrum. The bottom panel shows sonogram (FFT size 1024, Hann (cosine bell), window overlap 50%) with an example of wakefulness period marked by yellow rectangle.

2. *Slow-wave sleep* characterized by closed eyes and specific sleeping posture with particular muscle tone. The frontal left EEG showed mostly slow, high-amplitude activity (up to 1000 uV) at 0.5–4 Hz, with some 8–12 Hz sleep spindles mixed in (Figure S4.2).

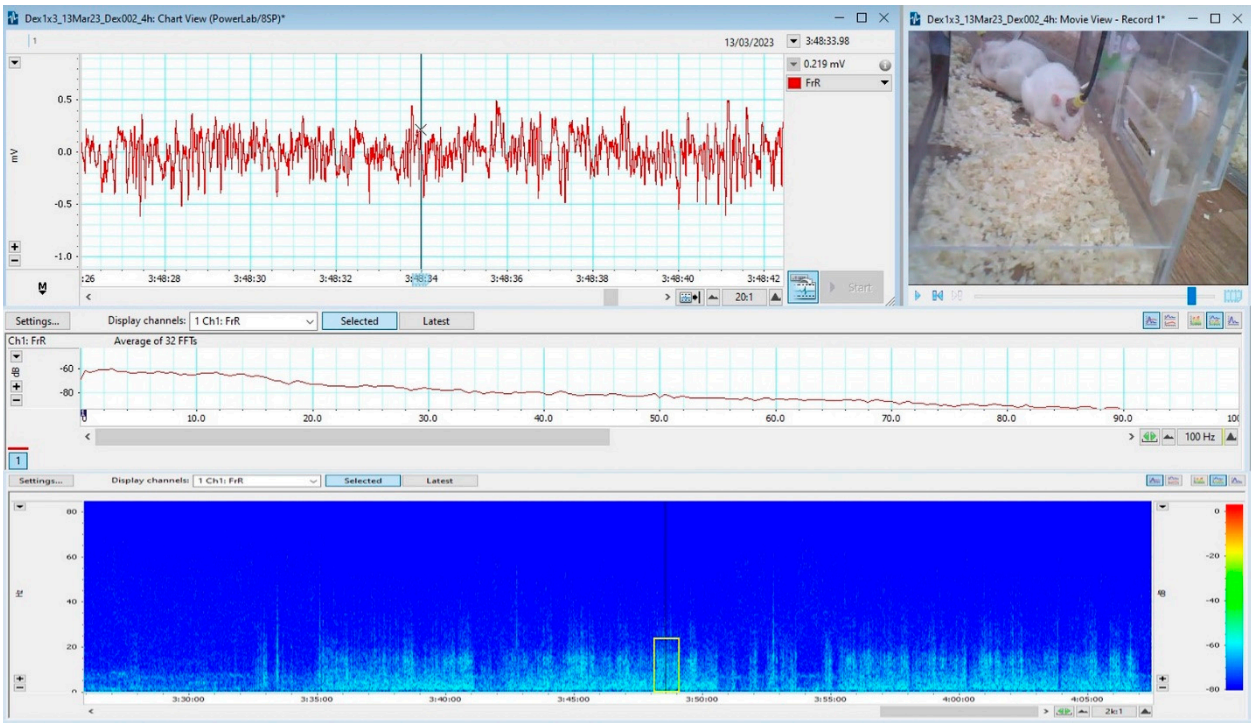

Figure S4.2. Slow-wave sleep state. The upper panel shows frontal left EEG with vertical blue line corresponding to video screenshot on the right (typical sleeping posture). The middle panel shows power-spectrum of frontal left EEG during slow-wave sleep: low delta frequencies (0.5-4 Hz) are predominant, and delta power exceeds that in wakefulness. The bottom plot shows sonogram (FFT size 1024, Hann (cosine bell), window overlap 50%) with an example of slow-wave sleep period marked by yellow rectangle. Frequencies between 10 and 20 Hz are more prominent than during wakefulness shown in Figure S4.1.

3. *Spike-wave epileptic activity* was associated with behavioral arrest and immobility. High-voltage (1200–2000 uV) repetitive spikes at 7–8 Hz were present in the frontal left EEG (Figure S4.3). This epileptic activity was also identified automatically as spike-wave discharges, SWDs (Supplementary S1). Dex injections enhanced spike-wave activity with numerous discontinuous SWDs interrupted by non-SWD activity lasting from several seconds to several minutes. For the analysis of behavioral states dynamics, long periods containing SWDs were marked as ‘spike-wave epileptic activity’ if time intervals between successive SWDs were less than 5 minutes.

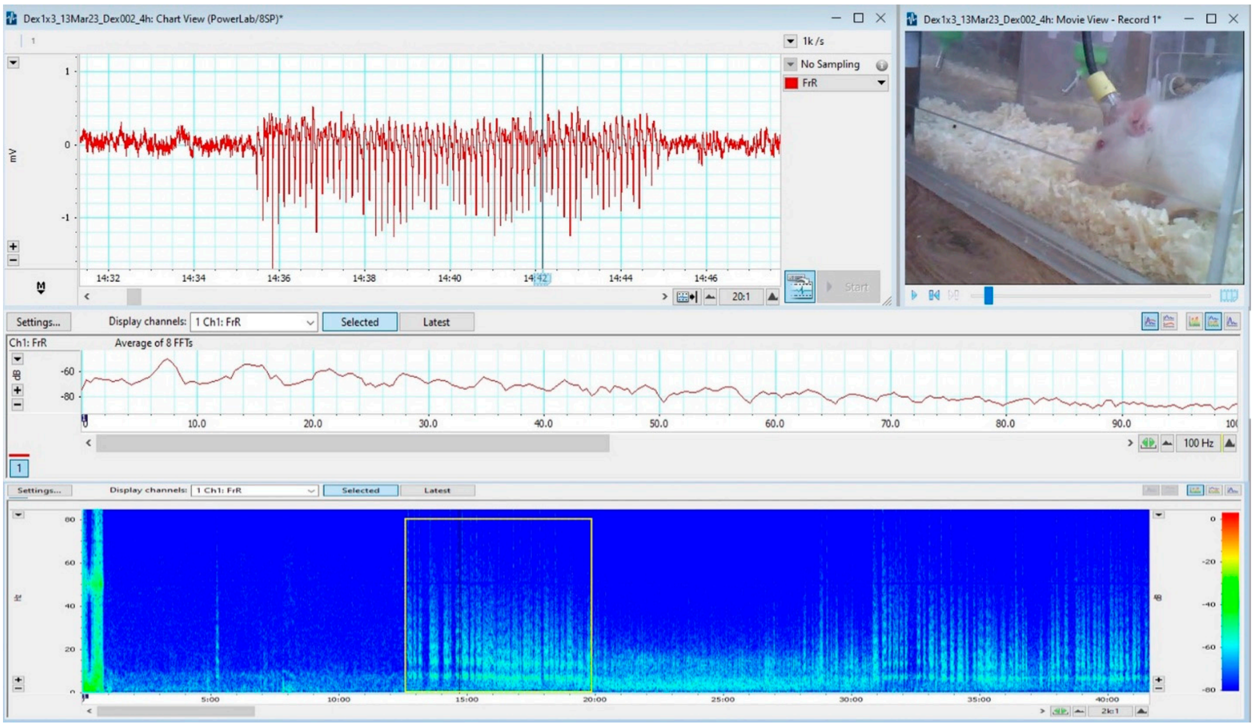

Figure S4.3. Spike-wave epileptic activity. The upper panel shows frontal left EEG with vertical blue line corresponding to video screenshot on the right (behavioral arrest). The middle panel shows power-spectrum of frontal left EEG during spike-wave epileptic activity: the main peak on 6-7 Hz and additional peaks on multiple frequencies. The bottom plot shows sonogram (FFT size 1024, Hann (cosine bell), window overlap 50%) with one of spike-wave epileptic activity periods labeled by yellow rectangle.

4. *Deep sedation phase*. Periods with very low muscle tonus (i.e. rat was lying flat on the stomach) and eyes opened. The EEG showed mostly slow (0.5–4 Hz), high-amplitude activity (up to 1000 uV). There was just slight modulation of the frontal left EEG signal amplitude (Figure S4.4).

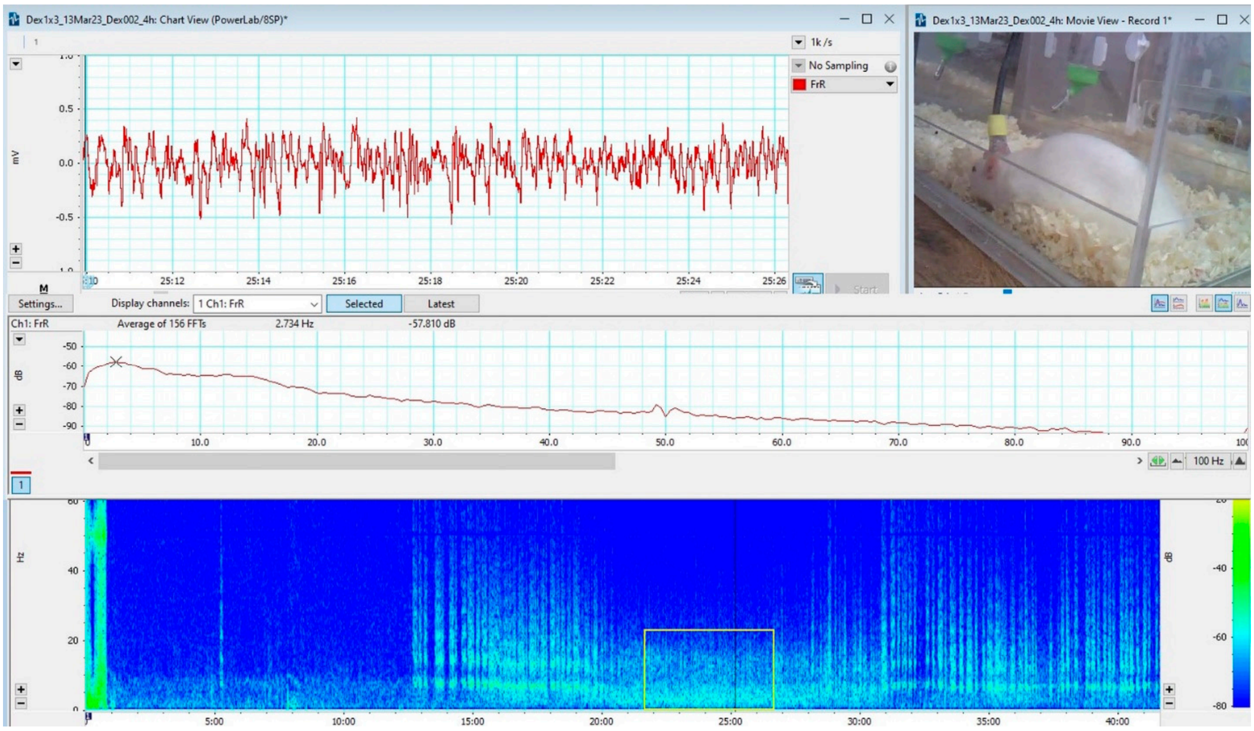

Figure S4.4. Deep sedation state. The upper panel shows frontal left EEG with vertical blue line corresponding to video screenshot on the right (lying flat on the stomach, eyes opened); high amplitude slow waves could be seen. The middle panel shows power-spectrum of frontal left EEG during deep sedation phase: low delta frequencies (0.5-4 Hz) are prevailing. The bottom plot shows sonogram (FFT size 1024, Hann (cosine bell), window overlap 50%) with the deep sedation state period labeled by yellow rectangle.

5. *State with mixed EEG patterns* (Figure S4.5). Periods showing mixed behavioral and EEG patterns of deep sedation phase and spike-wave epileptic activity: very low muscle tonus (animal is lying flat on its stomach) and eyes opened, slow delta waves predominance in the frontal left EEG intermitted with the epileptiform activity (spikes and sharp waves, but not mature SWDs).

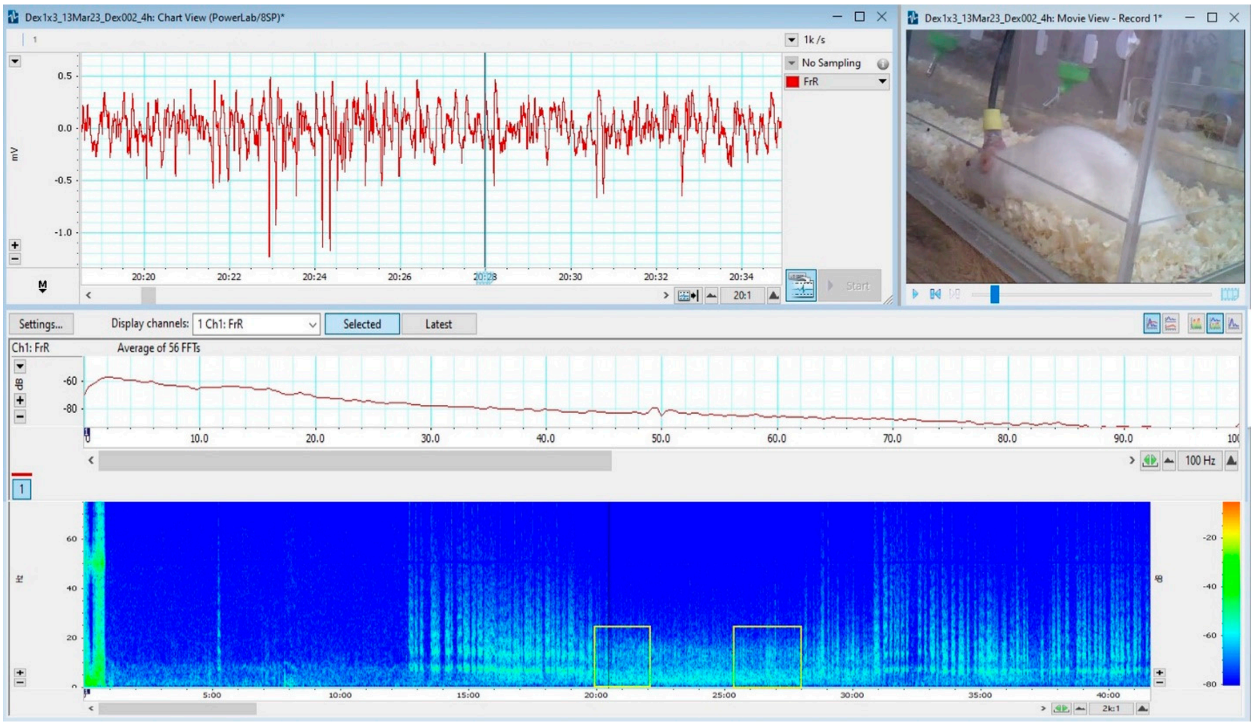

Figure S4.5. The state with mixed EEG patterns. The upper panel shows frontal left EEG with vertical blue line corresponding to video screenshot on the right (lying flat on the stomach, eyes opened). In this plot, slow waves are mixed with sharp spikes. The middle plot shows power-spectrum of frontal left EEG during the state with mixed EEG. The bottom plot shows sonogram (FFT size 1024, Hann (cosine bell), window overlap 50%), in which two periods of mixed EEG patterns are marked by yellow rectangles: the first is at the beginning of sedation state, and the second is at the end; the period between the rectangles corresponds to the deep sedation phase.
